# Enhancing Health for the Growing Number of Older Cancer Survivors: Designing Innovative Behavioral Interventions

**DOI:** 10.1093/geroni/igaa057.2238

**Published:** 2020-12-16

**Authors:** Heather Derry, Claire Conley, Kelly Trevino

## Abstract

By 2040, there will be an estimated 26.1 million cancer survivors in the United States, with 73% over age 65. Compared to younger survivors and those without cancer, older adult cancer survivors have an elevated comorbidity burden. Lifestyle interventions can play a key role in preventing and managing chronic health conditions and promoting quality of life during and after cancer treatment. Yet, behavioral interventions for maximizing health are under-utilized in older adults with cancer. At times, older adults may have unique needs that require tailoring to increase accessibility, optimization, and uptake of behavioral interventions.

This symposium will showcase innovative approaches for enhancing health among older adult cancer survivors during and after cancer treatment. Dr. Bluethmann will discuss design considerations for using geriatric assessment in an ongoing exercise trial to manage side effects of aromatase inhibitors. Dr. Gell will present data on older survivors’ preferences regarding text messaging to support physical activity maintenance from an intervention study. Dr. Leach will discuss the use of technology to facilitate lifestyle change in older cancer survivors, presenting data on older adults’ user preferences and benefits from an eHealth tool. The discussant, Dr. Trevino, will summarize how these interventions can be leveraged to promote engagement in managing older survivors’ health and to inform next steps in intervention development. Collectively, this multidisciplinary group of speakers will provide practical information and “lessons learned” from designing behavioral and technology-based interventions, and highlight the promise that these approaches hold for improving quality of life in aging cancer survivors. Cancer and Aging Interest Group Sponsored Symposium.

